# Deletion of the Neurotrophic Factor neudesin Prevents Diet-induced Obesity by Increased Sympathetic Activity

**DOI:** 10.1038/srep10049

**Published:** 2015-05-08

**Authors:** Hiroya Ohta, Morichika Konishi, Yusuke Kobayashi, Atsuki Kashio, Takayuki Mochiyama, Shigenobu Matsumura, Kazuo Inoue, Tohru Fushiki, Kazuwa Nakao, Ikuo Kimura, Nobuyuki Itoh

**Affiliations:** 1 Department of Genetic Biochemistry, Kyoto University Graduate School of Pharmaceutical Sciences, Kyoto, Japan; 2 Department of Microbial Chemistry, Kobe Pharmaceutical University, Kobe, Japan; 3 Laboratory of Nutrition Chemistry, Division of Food Science and Biotechnology, Graduate School of Agricultures, Kyoto University, Kyoto, Japan; 4 Medical Innovation Center, Kyoto University Graduate School of Medicine, Kyoto, Japan; 5 Department of Applied Biological Science, Graduate School of Agriculture, Tokyo University of Agriculture and Technology, Tokyo, Japan; 6 Department of Pharmacogenomics, Kyoto University Graduate School of Pharmaceutical Sciences, Kyoto, Japan

## Abstract

Some neurotrophic factors, which are potent regulators of neuronal development and function, have recently been implicated in the control of energy balance by increasing energy expenditure. We previously identified neudesin as a novel neurotrophic factor with potential roles in the central nervous system. Although *neudesin* is also expressed in various peripheral tissues including adipose tissue, its physiological roles have not yet been elucidated. We found that *neudesin* knockout (KO) mice were resistant to high-fat diet-induced obesity and obesity-related metabolic dysfunctions. *neudesin* KO mice exhibited increased energy expenditure due to increased sympathetic activity, which resulted in increased heat production and fatty acid oxidation in brown adipose tissue and enhanced lipolysis in white adipose tissue. Thus, neudesin, which may be a negative regulator of sympathetic activity, could represent a novel regulator of the development of obesity and obesity-related metabolic dysfunctions.

The epidemic of obesity is closely associated with an increase in metabolic disorders, including diabetes, dyslipidemia, and cardiovascular diseases^1^,^2^,^3^, and has, thus, propelled major interest in the regulation of adipose functions. Adipose tissue can be divided into two distinct types: white and brown. White adipose tissue (WAT) is specialized for the storage of excess energy as triglycerides (TG)^4^, whereas brown adipose tissue (BAT) dissipates energy as heat, thereby counteracting obesity^5^. The sympathetic nervous system (SNS) plays fundamental roles in maintaining energy homeostasis in living organisms^6^. The SNS is essential for regulating adipose function and the development of obesity because it stimulates lipolysis in WAT and enhances heat production in BAT by activating adrenergic signaling^7^,^8^,^9^.

Neurotrophic factors promote the survival, differentiation, and maintenance of neurons in the vertebrate nervous system and are crucial for nervous system functions^10^,^11^,^12^. Some neurotrophic factors have recently been recognized as crucial regulators of energy metabolism^13^,^14^. For example, the administration of brain-derived neurotrophic factor (BDNF) was shown to increase energy expenditure^15^, and *Bdnf* heterozygous mice exhibited obesity phenotypes^16^. The administration of ciliary neurotrophic factor, another neurotrophic factor, suppressed adiposity, hyperphagia, and hyperinsulinemia in *ob/ob* mice^17^. Thus, neurotrophic factors generally induce anti-obesity effects by suppressing food intake or increasing energy expenditure.

We previously identified the neurotrophic factor, neudesin, which was well conserved in various vertebrates, including humans, mice, and zebrafish^18^,^19^,^20^. Previous studies reported that *neudesin* was expressed in the brain and spinal cord of embryonic mice and appears to be essential for neuronal function^18^,^19^,^20^,^21^,^22^. The administration of recombinant neudesin to the hypothalamus was reported to repress appetite^24^. Novais *et al.* generated *neudesin* KO mice and showed that the *neudesin* deletion resulted in shorter dendrites in the dentate gyrus of the hippocampus and increased anxious-like behaviors^25^. This is the first and only study that described the physiological role of neudesin in the central nervous system (CNS); however, its systemic roles are not yet fully understood. *neudesin* was also expressed in various peripheral tissues in adult mice, including adipose tissue, and several studies suggested that it may play roles in peripheral tissues^19^,^20^,^23^. Thus, determining the system effects of *neudesin* in various tissues is essential for elucidating its physiological effects. Therefore, we also generated *neudesin* KO mice that were resistant to high-fat diet (HFD)-induced obesity and obesity-related metabolic dysfunctions. The present findings represent the first report that a neurotrophic factor suppresses energy expenditure. neudesin, which may be a negative regulator of sympathetic activity, could represent a novel regulator of the development of obesity and obesity-related metabolic dysfunctions.

## Results

### *neudesin* KO mice were resistant to diet-induced obesity

**Figure 1:**
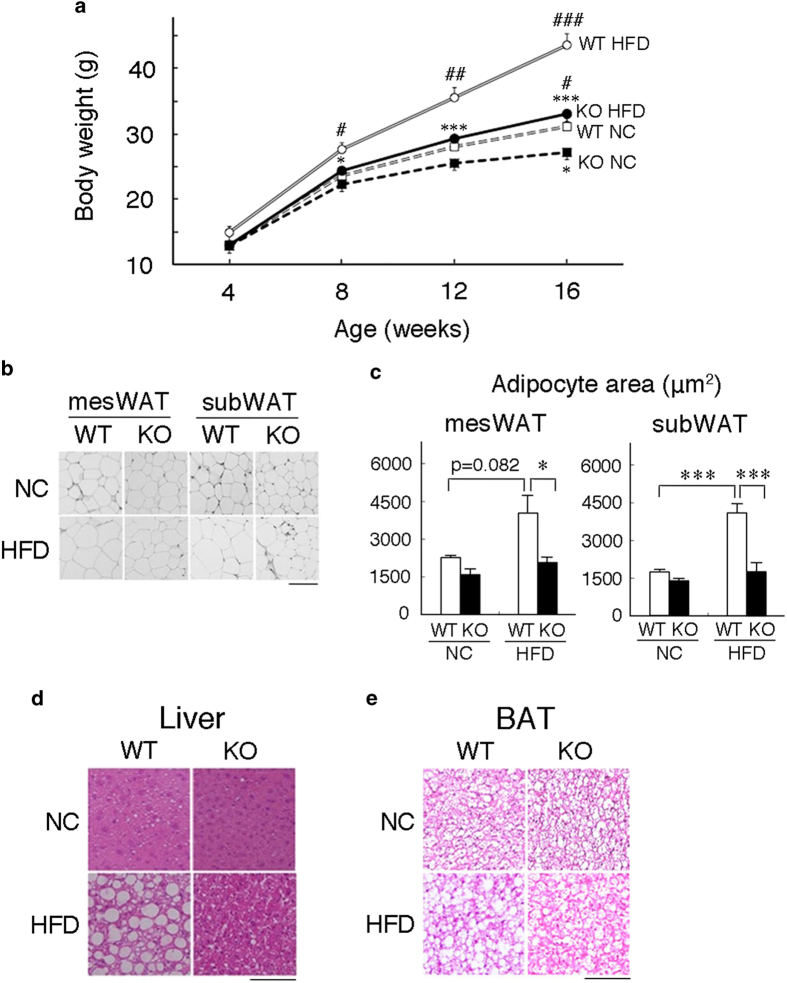
***neudesin* KO mice were resistant to diet-induced obesity** (**a**): Body weight changes in wild type (WT) and *neudesin* knockout (KO) mice fed normal chow (NC) or high-fat diet (HFD). All mice were weighed weekly (n = 6–14; *, p < 0.05, ***, p < 0.001 vs. WT within the same condition. #, p < 0.05, ##, p < 0.01, ###, p < 0.001 vs. feeding within the same genotype). (**b**) Paraffin sections of mesenteric white adipose tissue (mesWAT) and subcutaneous WAT (subWAT) from WT and *neudesin* KO mice at 16 weeks of age were stained with hematoxylin and eosin. Scale bar, 100 μm. (**c**) The adipocyte areas of WT and *neudesin* KO mice at 16 weeks of age. The average adipocyte cell areas were measured using at least 100 cells per mouse (n = 3–5; *, p < 0.05; ***, p < 0.001). (**d**) Frozen sections of the liver of WT and *neudesin* KO mice fed a NC or HFD at 16 weeks of age were stained with hematoxylin and eosin. Scale bar, 100 μm. (**e**) Paraffin sections of brown adipose tissue (BAT) of WT and *neudesin* KO mice at 16 weeks of age were stained with hematoxylin and eosin. Scale bar, 100 μm.

*neudesin* was previously reported to be expressed in the CNS and various peripheral tissues of adult mice^18^,^19^,^20^,^23^ (Fig. S1A). We generated *neudesin* KO mice to elucidate its physiological roles. *neudesin* KO mice showed the expected Mendelian ratios (Fig. S1B-G) and were apparently normal and fertile. We then examined the body weights and tissue weights of WT and *neudesin* KO mice. Body weights at 8 weeks of age and E18.5 were similar between wild type (WT) and *neudesin* KO mice. The weights of principal tissues including the liver, heart and kidney but not WAT at 8 weeks of age were also similar between WT and *neudesin* KO mice (Fig. 1A, Fig. S2 and Table. S1). Tibia lengths, which reflected growth^26^, were also similar between WT and *neudesin* KO mice (Fig. S1H). These results suggested that the growth of *neudesin* KO mice was essentially normal. However, increases in body weight were suppressed in *neudesin* KO mice as they grew, and at 16 weeks of age, their body weights were lower than those of WT mice. Increases in body weights due to HFD in WT mice were strongly suppressed in *neudesin* KO mice (Fig. 1A).

We measured the weights of various tissues at 16 weeks of age and the obtained results were summarized in Table 1. The WAT weight was significantly lower in *neudesin* KO mice than in WT mice when they were fed normal chow (NC). When WT mice were fed HFD, WAT weight was greater than when fed NC, whereas this increase was strongly suppressed in *neudesin* KO mice (Table 1). We next examined the morphology of white adipocytes (Fig. 1B). The sizes of white adipocytes in *neudesin* KO mice fed NC were not significantly different from those in WT mice fed NC. HFD caused adipocyte hypertrophy in WT mice, which was significantly suppressed in *neudesin* KO mice (Fig. 1C).

The weights of the liver and BAT were similar between WT and *neudesin* KO mice when they were fed NC (Table 1). Increases were observed in the weights of the liver and BAT of WT mice fed HFD, whereas these increases were significantly less in *neudesin* KO mice fed HFD. We then histologically examined the liver and BAT of WT and *neudesin* KO mice (Fig. 1D,E). The liver was apparently normal in both WT and *neudesin* KO mice fed NC, whereas the marked accumulation of lipids was observed in WT mice fed HFD. However, HFD-induced lipid accumulation in the liver was significantly decreased in *neudesin* KO mice (Fig. 1D). BAT was also apparently normal in both WT and *neudesin* KO mice fed NC, whereas the formation of large lipid droplets was observed in WT mice fed HFD. The formation of lipid droplets was suppressed in *neudesin* KO mice (Fig. 1E). The weights of other tissues examined were similar between WT and *neudesin* KO mice. These results suggested that *neudesin* KO mice were resistant to diet-induced obesity (DIO).

### *neudesin* KO mice were protected from obesity-induced metabolic dysfunctions

We measured various metabolic parameters and the results were summarized in Table 2. The levels of glucose, total cholesterol, and leptin were significantly increased in WT mice fed HFD, and this increase in metabolic parameters was generally suppressed in *neudesin* KO mice fed HFD. These results suggested that *neudesin* KO mice were protected from obesity-induced metabolic dysfunctions.

**Figure 2:**
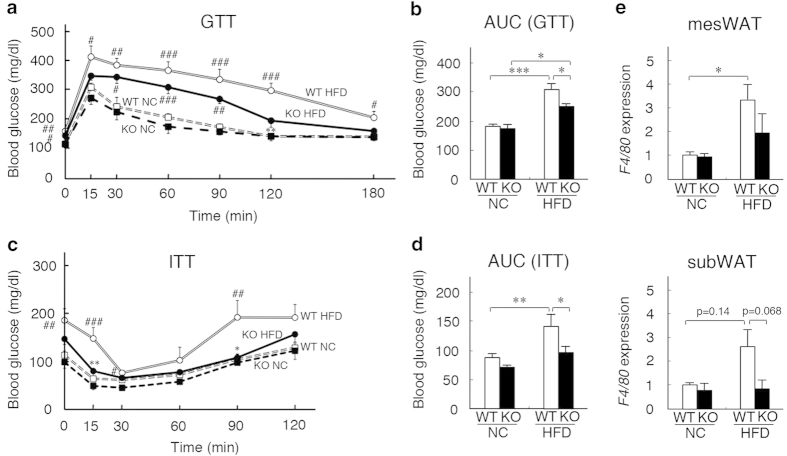
***neudesin* KO mice were resistant to obesity-induced metabolic dysfunctions** (**a**): The results of the glucose tolerance test (GTT) on WT mice and *neudesin* KO mice fed NC or HFD at 16 weeks of age (n = 6–7; **, p < 0.01 vs. WT within the same condition. #, p < 0.05, ##, p < 0.01, ###, p < 0.001, vs. feeding within the same genotype). The blood glucose levels at an initial point were 114.3 ± 6.5 mg/dl for WT NC, 111.3 ± 9.1 mg/dl for KO NC, 157.3 ± 7.1 mg/dl for WT HFD and 137.8 ± 15.0 mg/dl for KO HFD. (**b**) The average area under the blood glucose curve (AUC) for GTT (n = 6–7; *, p < 0.05, ***, p < 0.001). (**c**) The results of the insulin tolerance test (ITT) on WT and *neudesin* KO mice fed NC or HFD at 16 weeks of age (n = 3–7; *, p < 0.05, **, p < 0.01 vs. WT within the same condition. #, p < 0.05, ##, p < 0.01, ###, p < 0.001, vs. feeding within the same genotype). The blood glucose levels at an initial point were 111.1 ± 8.5 mg/dl for WT NC, 107.3 ± 9.8 mg/dl for KO NC, 184.0 ± 17.2 mg/dl for WT HFD and 145.8 ± 11.5 mg/dl for KO HFD. (**d**) The average area under the blood glucose curve (AUC) for ITT (n = 3–7; *, p < 0.05, **, p < 0.01). (**e**) The expression levels of F4/80 in mesenteric WAT and subcutaneous WAT of WT and *neudesin* KO mice fed a NC or HFD at 16 weeks of age. *18S* rRNA levels were used as an internal control and expression levels in WT mice fed NC were taken as 1.0 (n = 3–5; *, p < 0.05).

Obesity is known to cause insulin resistance. Blood glucose and plasma insulin levels were similar between WT and *neudesin* KO mice when fed NC (Table 2). WT mice showed increased glucose and insulin levels when fed HFD, whereas this HFD-induced increase in glucose and insulin levels was suppressed in *neudesin* KO mice (Table 2). These results indicated that *neudesin* KO mice were protected from insulin resistance. We then performed glucose tolerance and insulin tolerance tests. Glucose tolerance in WT mice was impaired by HFD and this effect was significantly improved in *neudesin* KO mice (Fig. 2A,B). In parallel, insulin sensitivity was aggravated in WT mice fed HFD, but was also significantly improved in *neudesin* KO mice (Fig. 2C,D). These results suggested that *neudesin* KO mice were protected from HFD-induced insulin resistance.

Obesity can also induce the infiltration of macrophages to adipose tissue, which causes inflammation. We examined the expression levels of *F4/80*, a macrophage marker^27^. The expression levels of *F4/80* in WT mice fed HFD were significantly higher in mesenteric WAT and tended to be higher in subcutaneous WAT compared with those in WT mice fed NC. However, its expression levels in *neudesin* KO mice fed HFD were not significantly but tended to be lower than those in WT mice fed HFD (Fig. 2E). Thus, *neudesin* KO mice might be protected from obesity-induced inflammation.

### Energy expenditure was increased in *neudesin* KO mice

We found that *neudesin* KO mice were resistant to DIO and obesity-induced metabolic dysfunctions including diabetes. We attempted to elucidate why *neudesin* KO mice were resistant to DIO. Food intake at 16 weeks of age was slightly but significantly lower in *neudesin* KO mice than in WT mice fed NC, whereas food intake at 16 weeks of age was similar between WT and *neudesin* KO mice fed HFD (Fig. S3A). Thus, resistance to DIO was independent of food intake. We estimated fat absorption by determining fecal weights and fecal TG contents and found that more than 99% TG in HFD was absorbed in both WT and *neudesin* KO mice (Fig. S3B). Thus, the deletion of *neudesin* had little effect on fat absorption.

**Figure 3:**
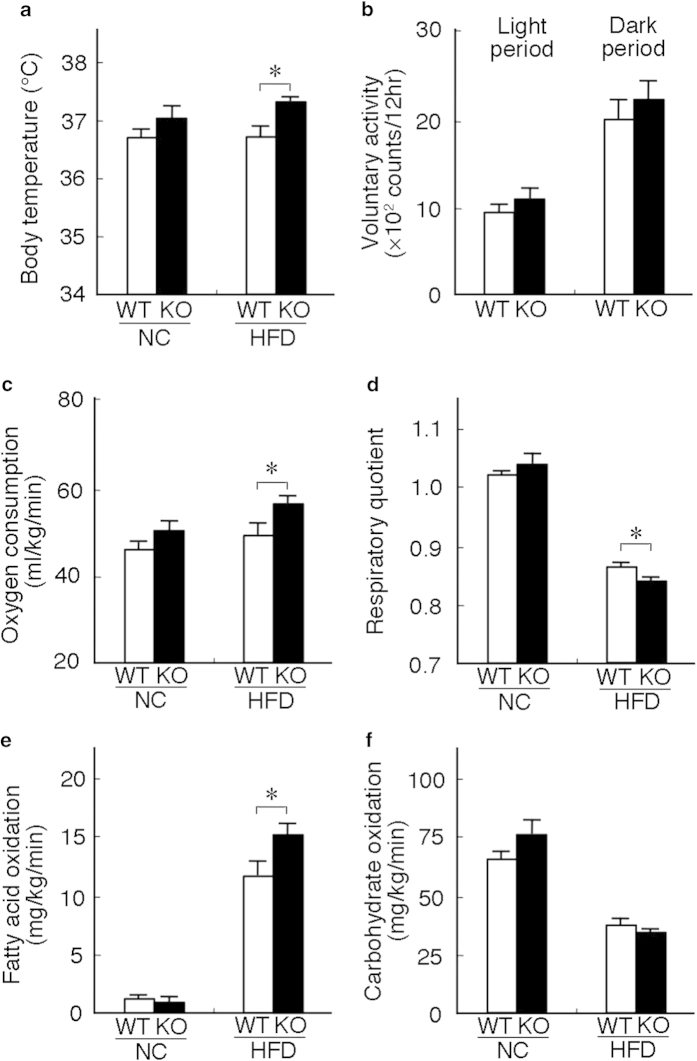
***neudesin* KO mice showed a systemic increase in energy expenditure** (**a**): Body temperature of WT and *neudesin* KO mice at 16 weeks of age (n = 4–6; *, p < 0.05). (**b**) The voluntary activity of WT and *neudesin* KO mice fed HFD at 12 weeks of age (WT: n = 3, KO: n = 4). Respiratory gas analysis performed on WT and *neudesin* KO mice at 12 weeks of age fed NC or HFD (n = 4–8; *, p < 0.05). (**c**) Oxygen consumption by WT and *neudesin* KO mice at 12 weeks of age. (**d**) The respiratory quotient (RQ) of WT and *neudesin* KO mice at 12 weeks of age. RQ was calculated based on O_2_ consumption and CO_2_ production. (**e**) Fatty acid oxidation by WT and *neudesin* KO mice at 12 weeks of age. (**f**) Carbohydrate oxidation by WT and *neudesin* KO mice at 12 weeks of age.

The enhancement observed in energy expenditure could have induced resistance to DIO. The rectal temperature of *neudesin* KO mice fed HFD was significantly higher than that of WT mice fed HFD, although voluntary activity was similar between WT and *neudesin* KO mice (Fig. 3A,B). Respiratory gas analysis showed that oxygen consumption was significantly increased in *neudesin* KO mice fed HFD (Fig. 3C). The respiratory quotient was significantly lower in *neudesin* KO mice fed HFD than in WT mice (Fig. 3D). Fatty acid oxidation was significantly increased in *neudesin* KO mice fed HFD (Fig. 3E). Carbohydrate oxidation was similar between WT mice and *neudesin* KO mice (Fig. 3F). These results indicated that energy expenditure in *neudesin* KO mice fed HFD was increased, which contributed to the observed resistance to DIO.

### Sympathetic activity was increased in *neudesin* KO mice

**Figure 4:**
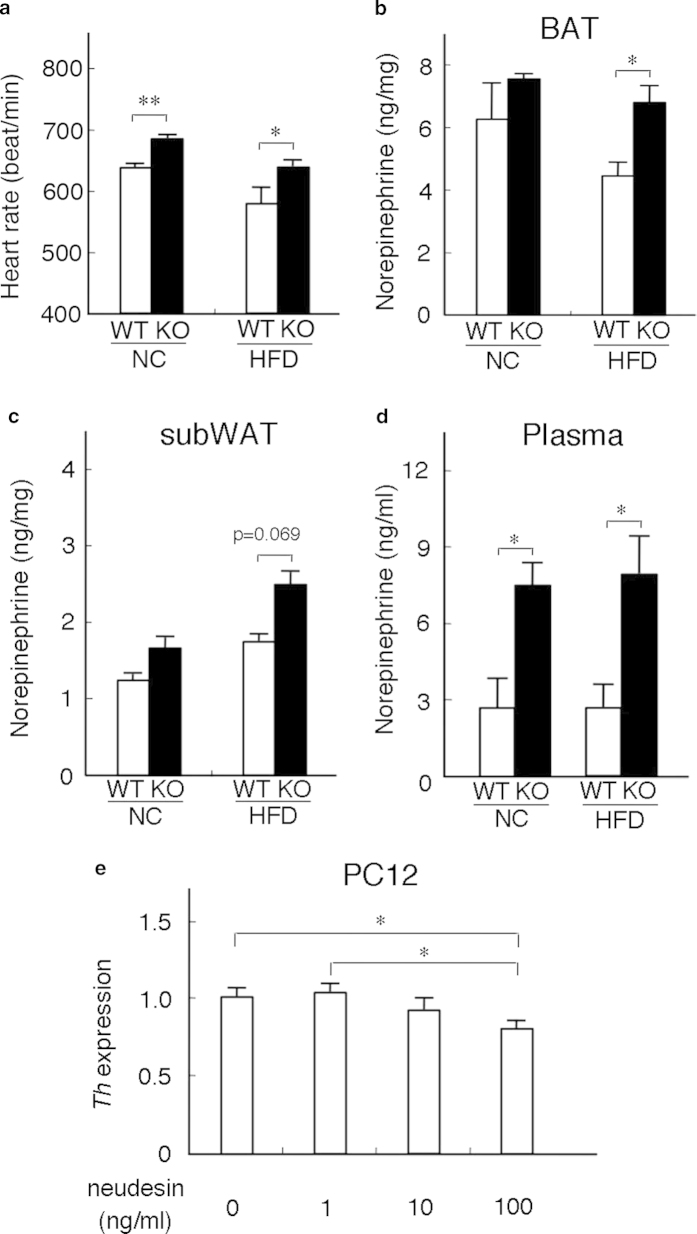
***neudesin* KO mice showed increased sympathetic activity** (**a**): The heart rates of WT and *neudesin* KO mice at 16 weeks of age (n = 4–6; *, p < 0.05; **, p < 0.01). (**b**) Norepinephrine levels in BAT of WT and *neudesin* KO mice at 16 weeks of age (n = 3–5; *, p < 0.05). (**c**) Norepinephrine levels in subcutaneous WAT of WT and *neudesin* KO mice at 16 weeks of age (n = 3–5). (**d**) Plasma levels of norepinephrine in WT and *neudesin* KO mice at 16 weeks of age (n = 4–5; *, p < 0.05). (**e**) The expression levels of *tyrosine hydroxylase (Th)* in differentiated PC12 cells (n = 5–6; *. p < 0.05). *18S* rRNA levels were used as an internal control and expression levels in cells without NGF and recombinant neudesin were taken as 1.0.

We attempted to elucidate why energy expenditure in *neudesin* KO mice was increased. As increased sympathetic activity often results in increased energy expenditure^6^,^28^, we examined the effects of the *neudesin* deletion on sympathetic activity. Heart rate, which is a common indicator of sympathetic activity^29^, was significantly increased in *neudesin* KO mice (Fig. 4A).

We next measured catecholamine levels in BAT and WAT, which also reflected sympathetic activity^30^. Norepinephrine levels were essentially similar in BAT and WAT of *neudesin* KO mice fed NC, whereas norepinephrine levels were significantly higher in BAT and tended to be higher in WAT of *neudesin* KO mice fed HFD (Fig. 4B,C). Norepinephrine levels in plasma were also significantly increased in *neudesin* KO mice fed NC and HFD (Fig. 4D). These results suggested that sympathetic activity was increased in *neudesin* KO mice fed HFD. In addition, we found that recombinant neudesin suppressed the expression levels of *tyrosine hydroxylase* (*Th*), which encodes a rate-limiting enzyme in the synthesis of norepinephrine, in differentiated PC12 cells, which show sympathetic neuron-like properties^31^ (Fig. 4E). Thus, neudesin may suppress sympathetic activity by reducing *Th* expression.

**Figure 5:**
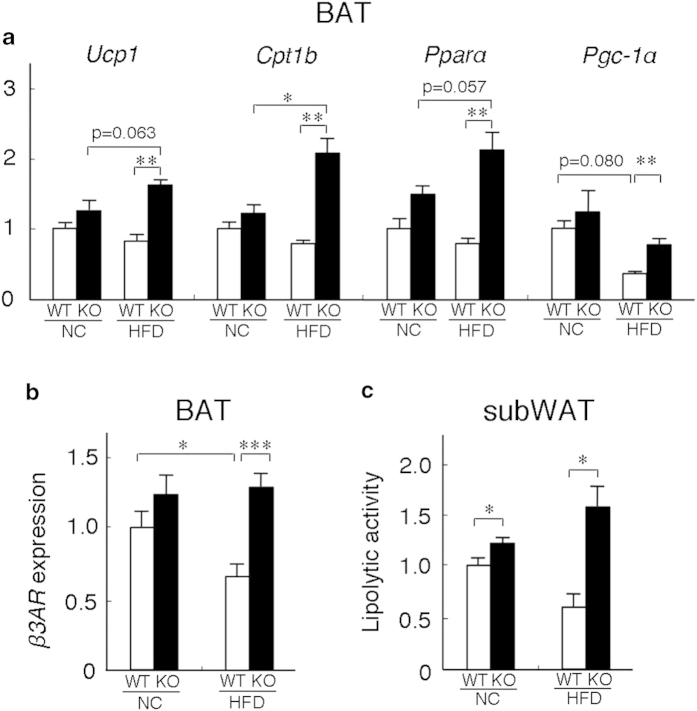
***neudesin* KO mice showed enhanced energy expenditure in BAT and WAT** (**a**): The expression levels of *Ucp1, Pgc-1α, Pparα*, and *Cpt1b* in the BAT of WT and *neudesin* KO mice at 16 weeks of age. *18S* rRNA levels were used as an internal control and expression levels in WT mice fed NC were taken as 1.0 (n = 5–7; *, p < 0.05; **, p < 0.01). (**b**) The expression levels of *β3AR* in the BAT of WT and *neudesin* KO mice at 16 weeks of age. 18 S rRNA levels were used as an internal control and expression levels in WT mice fed NC were taken as 1.0 (n = 5–7; *, p < 0.05, ***, p < 0.001). (**c**) Lipolytic activity in the subcutaneous WAT of WT and *neudesin* KO mice at 16 weeks of age. Lipolytic activity was standardized to that of WT mice fed NC and the lipolytic activity of WT mice fed NC was taken as 1.0 (n = 3–4; *, p < 0.05).

Enhanced sympathetic activity has been shown to stimulate β3-adrenergic receptors (*β3AR*) in adipose tissue^7^, increase heat production and fatty acid oxidation in BAT^9^, and promote lipolysis in WAT^8^. We then examined the physiological effect of this enhanced sympathetic activity on BAT and WAT. We firstly examined the expression levels of *Ucp1*, *Cpt1, Pparα*, and *Pgc-1α* in BAT. *Ucp1* is expressed exclusively in BAT and is indispensible for thermogenesis. CPT1 catalyzes the entry of fatty acids into mitochondria and is crucial for fatty acid oxidation. PPARα and PGC-1α are the transcriptional regulators of thermogenesis and fatty acid oxidation. The expression levels of *Ucp1*, *Cpt1, Pparα,* and *Pgc-1α* in BAT of *neudesin* KO mice at 4 and 16 weeks of age fed NC were similar to those in WT a mice (Fig. 5A and Fig. S4). However, these expression levels in BAT of *neudesin* KO mice at 16 weeks fed HFD were significantly higher than those in WT mice (Fig. 5A). We examined the expression levels of *β3AR* in BAT. Its levels in WT mice fed HFD were significantly lower than those in WT mice fed NC. However, its levels in *neudesin* KO mice fed HFD were similar to those in *neudesin* KO mice fed NC but significantly higher than those in WT mice fed HFD (Fig. 5B). The lipolytic activity of subcutaneous WAT in *neudesin* KO mice increased slightly when they were fed NC and increased markedly when they were fed HFD (Fig. 5C). These results indicated that sympathetic activity was increased in adipose tissue of *neudesin* KO mice. Thus, the energy expenditure was increased by the increased sympathetic activity in WAT and BAT of *neudesin* KO mice, resulting in the resistance to DIO. The hypothalamus greatly contributes to the regulation of sympathetic activity, and several neuropeptides including POMC, NPY, AGRP, BDNF, and CRF in the hypothalamus are the important regulators of sympathetic activity and affect the development of obesity^32^,^33^,^34^. To examine the roles of the neuropeptides in the hypothalamus in increased sympathetic activity in *neudesin* KO mice fed HFD, we examined the expression levels of *Pomc*, *Npy*, *Agrp*, *Bdnf*, and *Crf* in the hypothalamus. Their expression levels in *neudesin* KO mice fed HFD were essentially comparable to those in WT mice (Fig. S5). The results suggest that these neuropeptides did not contribute to the increased sympathetic activity observed in *neudesin* KO mice fed HFD.

## Discussion

In the present study, we generated *neudesin* KO mice to elucidate its physiological roles. The results obtained showed that *neudesin* KO mice were resistant to DIO and obesity-related metabolic dysfunctions. Energy expenditure in *neudesin* KO mice was increased by increased sympathetic activity. Thus, neudesin appeared to be a suppressor of energy metabolism in vivo.

neudesin was originally identified as a neurotrophic factor. Neurotrophic factors promote the survival, differentiation, and maintenance of neurons in the nervous system. Previous studies suggested that *neudesin* was expressed in the CNS and required for CNS functions and development^18^,^19^,^20^,^21^,^22^,^24^,^25^. For example, Novais *et al.* reported that *neudesin* KO mice exhibited increased anxious-like behaviors caused by shorter dendrites in the dentate gyrus of the hippocampus^25^. Byerly *et al.* also reported that *neudesin* was expressed in the paraventricular nuclei (PVN) and arcuate nucleus (ARC), which are important areas of the hypothalamus for regulating appetite^35^,^36^, and that the administration of recombinant neudesin into the hypothalamus repressed appetite^24^. However, we showed that food intake by *neudesin* KO mice fed NC was decreased, suggesting that neudesin increases food intake. This discrepancy may be explained by differences in the physiological analysis using *neudesin* KO mice and pharmacological analysis by the administration of recombinant neudesin. In contrast, as food intake was similar between WT and *neudesin* KO mice when fed HFD, resistance to DIO in *neudesin* KO mice was independent of food intake. Resistance to DIO in *neudesin* KO mice may be mediated by increased sympathetic activity. PVN and ARC also regulate energy expenditure by affecting sympathetic activity^37^,^38^. However, our results showed that the expression levels of hypothalamic neuropeptides, which are the important regulators of sympathetic activity and affect the development of obesity^32^,^33^,^34^, in *neudesin* KO mice fed HFD were similar to those in WT mice. Thus, increased sympathetic activity observed in *neudesin* KO mice fed HFD may not be mediated by hypothalamic regulation in the CNS. We found that recombinant neudesin might suppress *Th* expression in differentiated PC12 cells with sympathetic neuron-like properties^31^. Additionally, *neudesin* KO mice exhibited increased levels of norepinephrine in adipose tissue, which are good indicators of sympathetic activity^30^. Thus, peripheral neudesin may suppress sympathetic activity by suppressing *Th* expression in peripheral neurons. However, further studies are required to elucidate the roles of central and peripheral neudesin in regulating sympathetic activity.

*neudesin* was shown to be expressed in various peripheral tissues, including WAT and BAT. We could not detect neudesin in plasma (unpublished observation), indicating that it is a local signaling molecule in peripheral tissues. neudesin is expected to act on targeted cells through a specific neudesin receptor, which has not yet been identified^18^,^19^,^20^,^21^,^22^,^23^. neudesin in adipose tissue may suppress sympathetic activity by activating the neudesin receptor expressed in peripheral neurons in or near adipose tissue. The expression levels of *neudesin* in WAT were significantly increased in WT mice fed HFD, whereas its expression in BAT was essentially unchanged (Fig. S6). However, the precise mechanism underlying the increase in sympathetic activity in *neudesin* KO mice remains unknown. Further studies are required to elucidate the roles of neudesin in regulating sympathetic activity. Additionally, previous studies suggested that neudesin could act on non-neuronal cells besides neurons^23^,^39^. Thus, some of the functions of neudesin could not be explained by its neurotrophic factor properties. We need to examine the role of neudesin other than as a neurotrophic factor in the future to obtain a better understanding of its physiological roles.

Although neurotrophic factors generally increase energy expenditure, neudesin suppresses it. To the best of our knowledge, this is the first study to show that a neurotrophic factor suppresses energy expenditure. Thus, the present study may shed new light on the physiological roles of neurotrophic factors in energy metabolism. In conclusion, we showed that neudesin was a possible regulator of energy expenditure and may contribute to the development of obesity. *neudesin* KO mice were resistant to DIO and obesity-related metabolic dysfunctions, and exhibited increased energy expenditure. Increased energy expenditure was attributed to a systemic increase in sympathetic activity. Our results suggested the physiological importance of neudesin in regulating energy expenditure and its role as a suppressor of sympathetic activity. We expect that neudesin could represent a novel regulator of the development of obesity and obesity-related metabolic dysfunctions.

## Methods

### Gene Targeting

We produced knockout mice in which *neudesin* was absent in the whole body. Briefly, exons 2 and 3 of *neudesin*, which code the cytochrome b5-like heme/steroid-binding domain^22^, were deleted by targeting vectors including the neomycin resistance sequence. *neudesin* KO mice were created via the homologous recombinant ES cells of C57BL/6J mice. Male chimeras were mated with C57BL/6J female mice to obtain F1 *neudesin*^*+/−*^ mice. *neudesin*^*+/−*^ mice were maintained on a C57BL/6 background. Male and female *neudesin*^*+/−*^ mice were intercrossed to obtain WT and *neudesin*^*−/−*^ (*neudesin* KO) littermates. The genotypes of these mice were determined by polymerase chain reaction (PCR) using the following primers: P1 (5’-tttatggacgaggagccccctaca-3’), P2 (5’-agcgaaggagcaaagctgctattg-3’), and P3 (5’-ggccatcctggtctacctagaaat-3’). P1 and P3 produced a 229-bp fragment of the WT *neudesin* locus. P2 and P3 produced a 526-bp fragment of the mutant *neudesin* locus.

### Animal Experiments

All mice were maintained in a light-controlled room (lights on from 0800 to 2000 h). WT and *neudesin* KO mice were allowed free access to a normal diet (MF; 3.6 kcal/g, 12% kcal fat, source: soybean; Oriental Yeast, Tokyo, Japan) or high-fat diet (HFD; 5.24 kcal/g, 60% kcal fat, Research Diet, Inc., New Jersey, USA). The HFD-feeding experiments began at 4 weeks of age. All experiments were performed using male mice. Mice were weighed weekly and sacrificed to obtain tissues and plasma samples at 16 weeks of age. All animal studies were conducted in accordance with the International Guiding Principles for Biomedical Research Involving Animals, and approved by the Animal Research Committee of Kyoto University Graduate School of Pharmaceutical Sciences.

### RNA Isolation and Real-Time Quantitative RT-PCR

Total RNA was extracted from mouse tissues at 16 weeks of age using an RNeasy minikit (QIAGEN, Hilden, Germany). cDNA was synthesized from total RNA (1 μg) in a reaction mixture containing Moloney murine leukemia virus reverse transcriptase (Life Technologies, Maryland, USA) and a random hexadeoxynucleotide primer (Takara, Shiga, Japan). A quantitative RT-PCR (qRT-PCR) analysis was performed using a Thermal Cycler Dice (Takara) using SYBR qPCR Mix (TOYOBO, Osaka, Japan). *18S rRNA* levels were determined as an internal control. The primers used were 5’-agggagtggtgttcgatgtc-3’ and 5’-agccaacaatggggtact-3’ for *neudesin*, 5’-catcagccatgtgggtacag-3’ and 5’-agtgtaggaatcccgcaatg-3’ for *F4/80*, 5’-tgcctggcagatatcatcac-3’ and 5’-cagtttcggcaatccttctg-3’ for *Ucp1*, 5’-ccttccagtacaagcacggt-3’ and 5’-tgggtagcatagaggccctt-3’ for *Th*, 5’-aagagcgccgtgtgatttac-3’ and 5’-ccatcatcccgcagatttac-3’ for *Pgc-1α*, 5’-aatgcaattcgctttggaag-3’ and 5’-ggccttgaccttgttcatgt-3’ for *Pparα*, 5’-aggcctcgatgacaagaatg-3’ and 5’-ttccggaagagatcttggag-3’for *Cpt1b*, 5’-aagctacacgatgccatgtc-3’ and 5’-tctgtccctgtctgctgtttc-3’ for *β3AR*, 5’-gacacgtggaagatgccgag-3’ and 5’-cagcgagaggtcgagtttgc-3’ for *Pomc*, 5’-atgctgactgcaatgttgctg-3’ and 5’-cagacttagacctgggaactct-3’ for *Agrp*, 5’-aggcttgaagacccttccatg-3’ and 5’-gggtggaaacttggaaaagtcg-3’ for *Npy*, 5’-agagcagctgccttgatgtt-3’ and 5’-gtcgtcagacctctctcgaacc-3’ for *Bdnf*, 5’-gcagttagctcagcaagctcac-3’ and 5’-caaatgatatcggagctgcg-3’ for *Crf* and 5’-cgaacgtctgccctatcaactt-3’ and 5’-ccggaatcgaaccctgatt-3’ for *18S rRNA*^24^,^40^,^41^.

### Histological Analysis

The WAT and BAT of WT and *neudesin* KO mice at 16 weeks of age were fixed in Bouin’s fixative, dehydrated, embedded in paraffin, and sectioned. Sections (8μm) were stained with hematoxylin and eosin, and examined by light microscopy. Images of WAT sections were captured and adipocyte sizes were measured for at least 100 cells per mouse with Image J software (National Institutes of Health, Maryland, USA).

The unfixed livers of WT and *neudesin* KO mice at 16 weeks of age were frozen in optimum cutting temperature compound (Sakura Finetek, Tokyo, Japan). Frozen sections (16μm) were stained with hematoxylin and eosin.

### Biochemical Analysis

Blood glucose levels were measured with a Glutest R kit (Sanwa Kagaku Kenkyusho, Nagoya, Japan). Plasma insulin levels were measured using a mouse insulin ELISA kit (Morinaga, Tokyo, Japan). Plasma TG, non-esterified free fatty acid (NEFA), and total cholesterol levels were measured using the TG E-test, NEFA C-test, and cholesterol E-test (Wako, Osaka, Japan), respectively. Plasma leptin levels were measured using a mouse leptin ELISA kit (Morinaga). Norepinephrine, epinephrine, and dopamine levels in plasma, WAT, and BAT were measured by HPLC, as described previously^29^,^42^.

### Glucose Tolerance and Insulin Tolerance Tests

The glucose tolerance and insulin tolerance tests were performed at 16 weeks of age by an intraperitoneal injection of glucose (1.5 mg/g) or human regular insulin (1 units/kg) (MP Biomedicals, Ohio, USA) after 6 h of fasting. Blood samples were taken at different time points from a tail vein. Blood glucose and plasma insulin levels were measured, as described above.

### Measurement of Food Intake

Daily food intakes by WT and *neudesin* KO mice were measured at 16 weeks of age. The measurement of food intake was performed for 3 days and the average of 3 days was used in the analysis.

### Measurement of fecal TG levels

Fecal lipids were extracted from dried feces (50 mg) of WT and *neudesin* KO mice at 8 weeks of age using the Folch method and fecal triglyceride (TG) levels were measured using TG E-test.

### Respiratory Gas Analysis

Oxygen consumption by WT and *neudesin* KO mice was measured at 12 weeks of age with an indirect calorimetric system. Briefly, room air was pumped through an acrylic metabolic chamber, and subjected to gas analysis (model ARCO-2000; Arco System, Tokyo, Japan). The respiratory quotient, fatty acid oxidation, and carbohydrate oxidation were calculated based on O_2_ consumption and CO_2_ production^43^.

### Measurement of Heart Rates

Heart rates were measured in conscious mice at 16 weeks of age using a tail-cuff system (Softron, Tokyo, Japan)^29^. Heart rates were measured daily for at least 3 days and the average of the 3 days was used in the analysis.

### Production of Recombinant Mouse neudesin Protein

A recombinant neudesin protein was obtained as described previously^18^. Briefly, High Five cells infected with a recombinant baculovirus containing neudesin cDNA with His_6_ tag at the 3’ terminus of the coding region were cultured at 27 °C for 96 hr in Grace’s Insect Medium (Gibco, Rockville, MD, USA) with 10% fetal bovine serum (FBS). A recombinant neudesin protein was purified from the culture medium by affinity chromatography using Ni-NTA agarose (QIAGEN).

### Cultures of PC12 Cells

PC12 cells were cultured in Dulbecco’s modified Eagle’s medium (DMEM) (Gibco) supplemented with 10% horse serum (Gibco), 5% FBS, and 1% penicillin-streptomycin (Nacalai Tesque, Kyoto, Japan) at 37 °C. PC12 cells were seeded at 4.0 × 10^4^ cells/well on 24-well plates. Medium was replaced on the next day at the beginning of the culture, and NGF (50 ng/ml; alomone labs, Jerusalem, Israel) in DMEM supplemented with 1% FBS and 1% penicillin-streptomycin was added. NGF was removed 24 hrs later and recombinant neudesin in DMEM supplemented with 1% FBS and 1% penicillin-storeptomycin was added. Cells were collected 6hrs later and total RNA was extracted. The expression levels of *Th* were examined by qRT-PCR as described above.

### Measurement of Lipolytic Activity

The subcutaneous WAT of WT and *neudesin* KO mice at 16 weeks of age was homogenized in homogenizing buffer (50 mM Tris-HCl, pH8.0, 0.25 M sucrose, 1 mM EDTA, and 1% Protease Inhibitor Cocktail (Sigma-Aldrich, St. Louis, USA). Cell debris was removed by centrifugation at 1,000 g for 15 min to obtain cell extracts. The protein concentration in tissue extracts was determined using the Bio-Rad protein assay kit (Bio-Rad Laboratories, California, USA) with bovine serum albumin as the standard. The lipolytic activity of tissue extracts was determined using p-nitrophenyl laurate as the substrate^44^,^45^.

### Statistical Analysis

Data are expressed as means ± standard error of means (SEMs). The significance of differences in mean values was assessed with the Student’s *t*-test when two groups were analyzed. When more than two groups were compared, an analysis of variance was used to evaluate significant differences among groups, and if significant differences were confirmed, each difference was further examined by the Tukey-Kramer method. P < 0.05 was considered significant. GraphPad Prism6 was used for statistical analyse.

## Author Contributions

H.O., M.K., I.K. and N.I. designed the experiments. H.O., M.K., Y.K., A.K. and T.M. performed experiments. S.M., K.I., and T.F. performed respiratory gas analysis. S.M., K.I., T.F., and K.N. contributed to discussion. H.O., I.K. and N.I. wrote the paper. H.O., I.K. and N.I. are the guarantors of this work and have full access to all the data in the study and take responsibility for the integrity of the data and the accuracy of the data analysis.

## Additional Information

**How to cite this article**: Ohta, H. *et al.* Deletion of the Neurotrophic Factor neudesin Prevents Diet-induced Obesity by Increased Sympathetic Activity. *Sci. Rep.* **5**, 10049; doi: 10.1038/srep10049 (2015).

## Supplementary Material

Supplementary Information

## Figures and Tables

**Table 1:** Tissue weights of WT and *neudesin* KO mice fed NC and HFD at 16 weeks of age.

	**WT NC**	**KO NC**	**WT HFD**	**KO HFD**
mesenteric WAT (mg)	351.2 ± 29.6	204.3 ± 10.5 *	947.9 ± 160.4 #	303.4 ± 40.1 **
subcutaneous WAT (mg)	606.7 ± 60.8	390.6 ± 26.6	2022.4 ± 199.4 ###	792.9 ± 94.4 ***
epididymal WAT (mg)	715.8 ± 71.7	441.1 ± 39.5	2218.7 ± 101.6 ###	964.5 ± 116.6 ##, ***
BAT (mg)	86.0 ± 8.3	63.7 ± 6.5	155.8 ± 19.7 ##	99.2 ± 7.7 **
Liver (mg)	1198.5 ± 75.1	1095.3 ± 23.3	1422.6 ± 109.8	1001.9 ± 39.4 ***
Heart (mg)	132.7 ± 6.5	117.5 ± 4.0	137.2 ± 4.3	127.3 ± 3.8
Kidney (mg)	176.7 ± 6.0	155.6 ± 3.3	161.9 ± 4.6	155.2 ± 4.6
Spleen (mg)	69.2 ± 4.3	61.8 ± 5.5	120.2 ± 34.5	161.5 ± 23.2

Data are means ± standard error of means (SEMs) (n = 4–11. * p < 0.05, ** p < 0.01, *** p < 0.001, vs WT mice within the same condition. #, p < 0.05, ##, p < 0.01, ###, p < 0.001 vs. feeding within the same genotype).

**Table 2:** Metabolic parameters of WT and *neudesin* KO mice at 16 weeks of age.

	**WT NC**	**KO NC**	**WT HFD**	**KO HFD**
Glucose (mg/dl)	126.5 ± 7.4	112.0 ± 5.1	168.1 ± 7.4 #	135.2 ± 7.6 *
Insulin (ng/ml)	1.39 ± 0.20	0.95 ± 0.30	3.45 ± 0.51	0.79 ± 0.16 **
TG (mg/dl)	88.1 ± 10.4	74.5 ± 2.3	93.5 ± 5.5	60.2 ± 8.7
NEFA (mEq/L)	0.358 ± 0.032	0.365 ± 0.015	0.419 ± 0.019	0.293 ± 0.017 **
Total cholesterol (mg/dl)	69.6 ± 7.9	67.3 ± 5.7	231.3 ± 7.4 ###	153.0 ± 16.6 ###, **
Leptin (ng/ml)	1.6 ± 0.3	1.1 ± 0.2	28.7 ± 1.0 ###	14.5 ± 2.1 ###, ***

Data are means ± standard error of means (SEMs) (n = 4–9; *, p < 0.05, **, p < 0.01, ***, p < 0.001, vs WT mice within the same condition. #, p < 0.05, ###, p < 0.001 vs. feeding within the same genotype)
