# Supplementary material for: Deletion of the Neurotrophic Factor neudesin Prevents Diet-induced Obesity by Increased Sympathetic Activity
Source: Sci Rep. 2015 May 8;5:10049. doi: 10.1038/srep10049 (PMC4424804; doi:10.1038/srep10049)
Supplement: Supplementary Information [file srep10049-s1.pdf]

Deletion of the Neurotrophic Factor neudesin Prevents Diet-induced Obesity by Increased Sympathetic Activity

(Supplementary data)

Hiroya Ohta[1], Morichika Konishi[1, 2], Yusuke Kobayashi[1], Atsuki Kashio[1], Takayuki Mochiyama[1], Shigenobu Matsumura[3], Kazuo Inoue[3], Tohru Fushiki[3], Kazuwa Nakao[4], Ikuo Kimura[5, 6, *], and Nobuyuki Itoh[1, *]

[1]Department of Genetic Biochemistry, Kyoto University Graduate School of Pharmaceutical Sciences, Kyoto, Japan, [2]Department of Microbial Chemistry, Kobe Pharmaceutical University, Kobe, Japan [3]Laboratory of Nutrition Chemistry, Division of Food Science and Biotechnology, Graduate School of Agricultures, Kyoto University, Kyoto, Japan, [4]Medical Innovation Center, Kyoto University Graduate School of Medicine, Kyoto, Japan, [5]Department of Applied Biological Science, Graduate School of Agriculture, Tokyo University of Agriculture and Technology, Tokyo, Japan, [6]Department of Pharmacogenomics, Kyoto University Graduate School of Pharmaceutical Sciences, Kyoto, Japan

*Correspondence to: Ikuo Kimura (ikimura@cc.tuat.ac.jp) or Nobuyuki Itoh (itohnobu@pharm.kyoto-u.ac.jp)

Figure S1

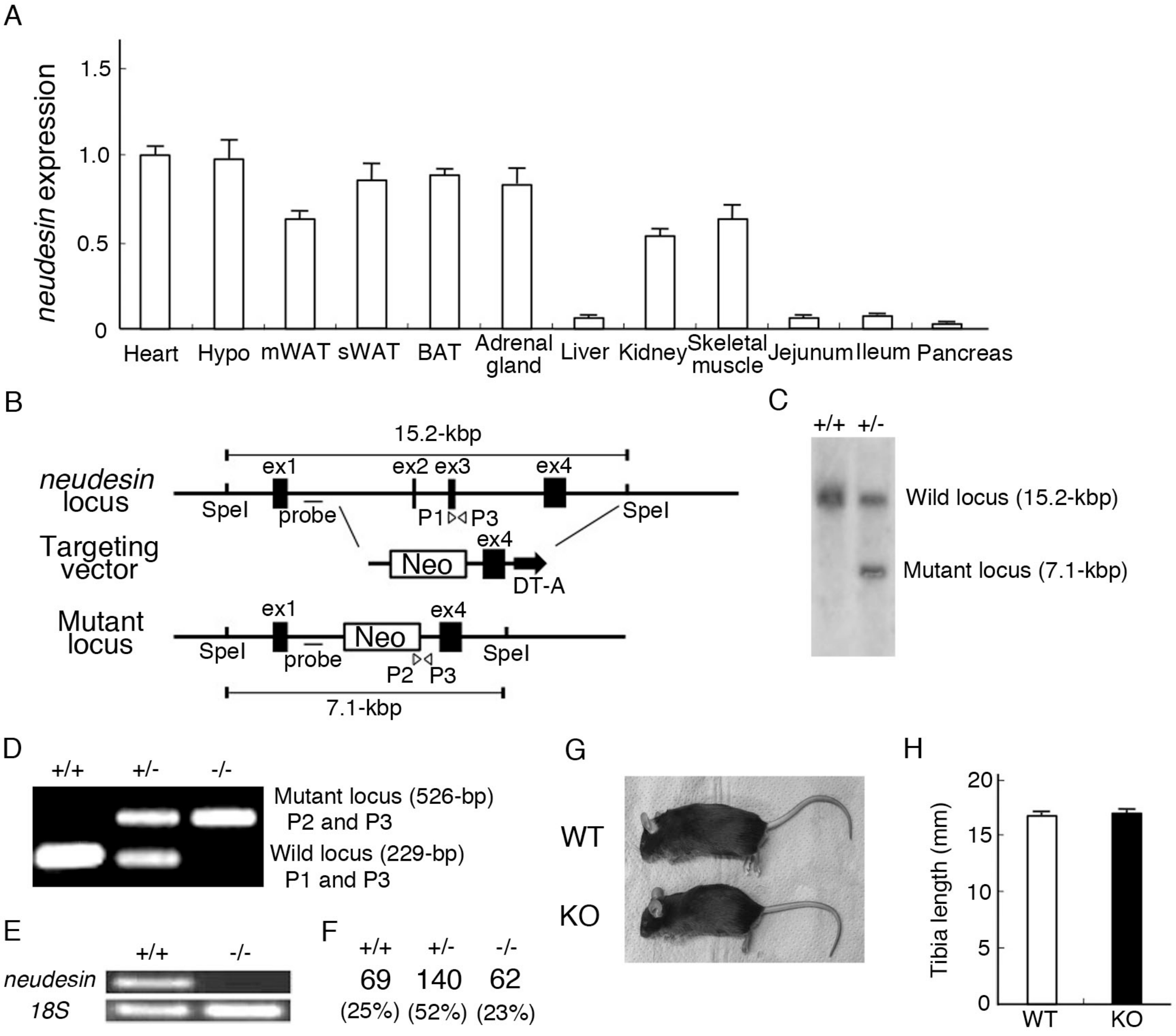
A
neudesin expression
1.5
1.0
0.5
0
Heart
Hypo
mWAT
sWAT
BAT
Adrenal gland
Liver
Kidney
Skeletal muscle
Jejunum
Ileum
Pancreas
B
15.2-kbp
neudesin locus
ex1
ex2
ex3
ex4
SpeI
probe
P1
P3
Targeting vector
Neo
DT-A
Mutant locus
P2
7.1-kbp
C
+/+
+/-
Wild locus (15.2-kbp)
Mutant locus (7.1-kbp)
D
-/-
Mutant locus (526-bp)
P2 and P3
Wild locus (229-bp)
P1 and P3
E
neudesin
18S
F
69
140
62
(25%)
(52%)
(23%)
G
WT
KO
H
Tibia length (mm)
20
15
10
5
0

Figure S1. The analysis of *neudesin* expression in adult mice and targeted disruption of *neudesin* in mice

A: The expression of *neudesin* in the heart, hypothalamus (hypo), mesenteric WAT, subcutaneous WAT, BAT, adrenal gland, liver, kidney, skeletal muscle, jejunum, ileum, and pancreas of mice at 16 weeks of age was measured by real-time quantitative RT-PCR using specific primers for mouse *neudesin*. *18S* rRNA levels were used as an internal control and expression levels in the heart were taken as 1.0 (n=3-5). B: A targeting vector was constructed by the ligation of three fragments. Exons 2 and 3 of *neudesin*, which code cytochrome b5-like heme/steroid-binding domain, were replaced by a neomycin resistance sequence. C: WT and *neudesin*-disrupted ES cells were examined by Southern blot analysis using a specific probe, as shown in Fig S1A. The 15.2-kb and 7.1-kb fragments, which corresponded to WT and neudesin mutant loci, respectively, were detected from the genomic DNA digested with Spe1. D: The genotypes of WT, *neudesin* heterozygous, and *neudesin* KO mice were determined by PCR using the three primers shown in the Research Design and Methods section. E: The expression levels of *neudesin* in the epididymal WAT of WT and *neudesin* KO mice at 8 weeks of age were examined by RT-PCR using the primers shown in the Research Design and Methods section. F: Mating heterozygous mice resulted in offspring with three genotypes at normal Mendelian ratios. G: Appearances of WT and *neudesin* KO mice at 16 weeks of age. *neudesin* KO mice appeared normal. H: Tibia lengths of WT and *neudesin* KO mice at 8 weeks of age (n=7-8). Tibia lengths were similar between WT and *neudesin* KO mice.

Figure S2

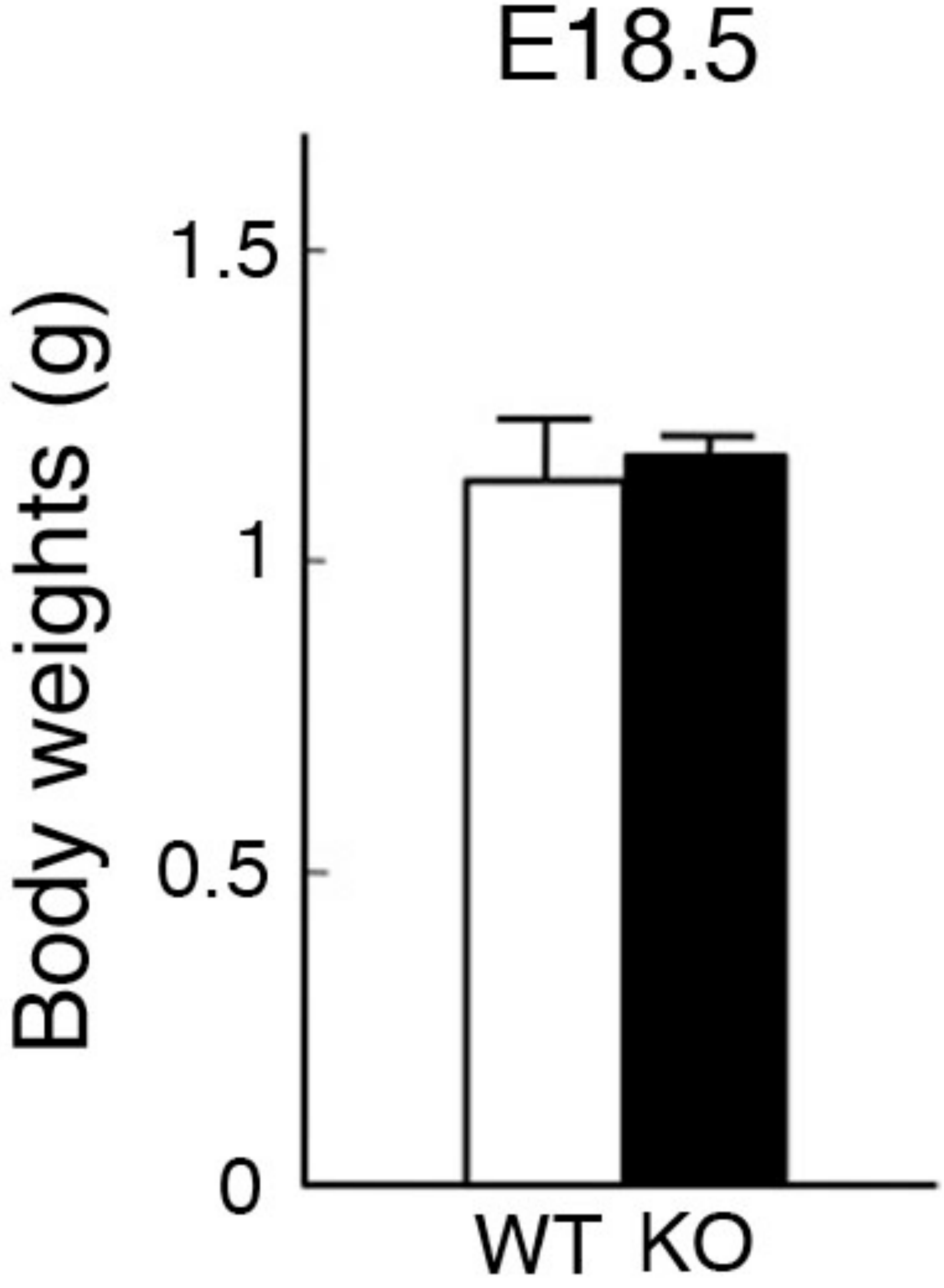

Figure S2. The body weights of WT and *neudesin* KO mice at embryonic day 18.5 (E18.5)

The body weights of WT and *neudesin* KO mice at E18.5 were measured (n=3-6).

Table S1: Tissue weights of WT and *neudesin* KO mice fed NC at 8 weeks of age

	**WT NC**	**KO NC**
**mesenteric WAT (mg)**	176.4±13.7	131.0±9.7 *
**subcutaneous WAT (mg)**	426.5±14.7	330.1±11.4 ***
**epididymal WAT (mg)**	337.8±20.4	276.8±8.7 *
**Liver (mg)**	1168.7±43.0	1064.5±34.3
**Heart (mg)**	122.6±5.5	117.6±4.7
**Kidney (mg)**	173.1±7.1	166.0±3.6

Data are means ± SEMs (n=9-10; * $p<0.05$, *** $p<0.001$, vs WT mice).

Figure S3

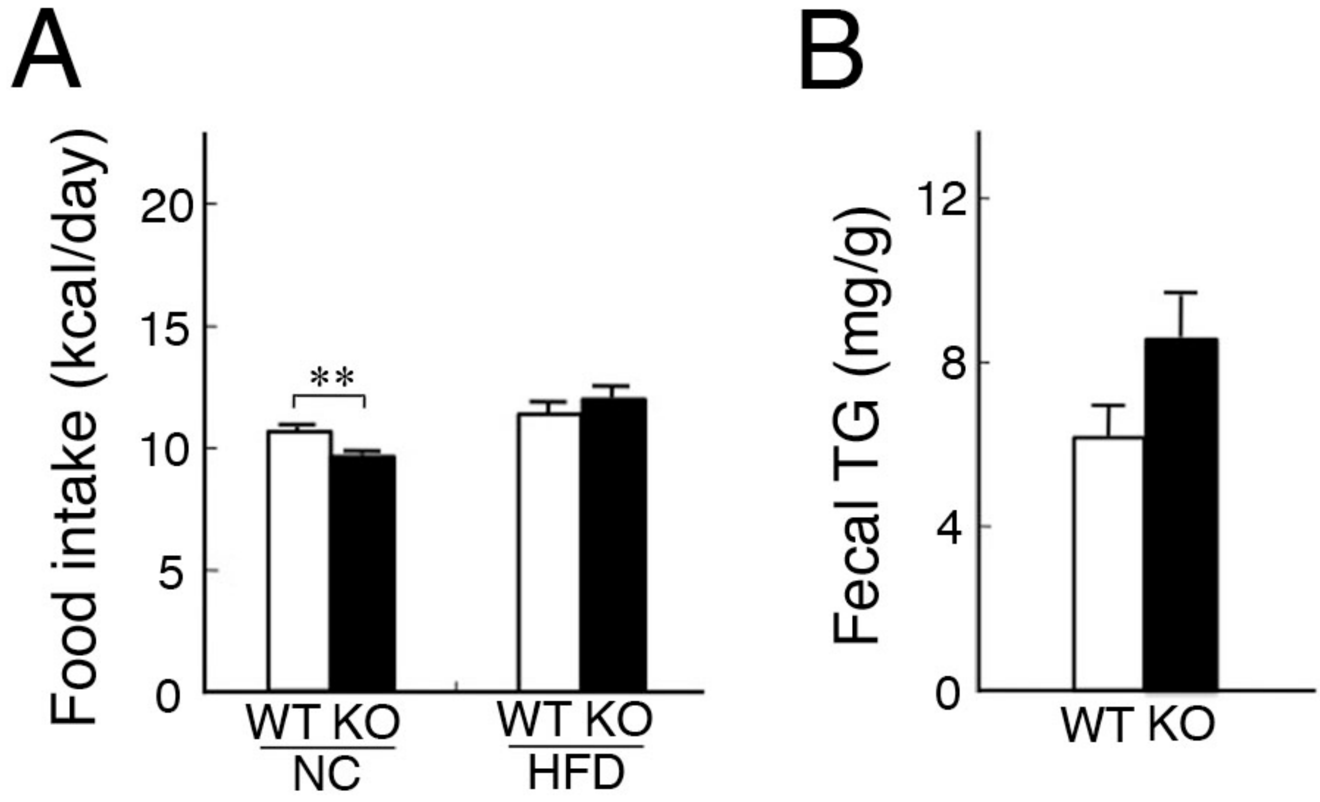
A
Food intake (kcal/day)
20
15
10
5
0
**
WT KO
NC
WT KO
HFD
B
Fecal TG (mg/g)
12
8
4
0
WT KO

Figure S3. Food intake and fecal TG levels in WT and *neudesin* KO mice

A: Daily food intake by WT and *neudesin* KO mice fed NC or HFD at 16 weeks of age was measured. The measurement of food intake was performed for 3 days and the average of 3 days was used in the analysis (n=6-10; **, $p<0.01$). B: Fecal TG levels of WT and *neudesin* KO mice fed HFD at 8 weeks of age were measured (n=4-5).

Figure S4

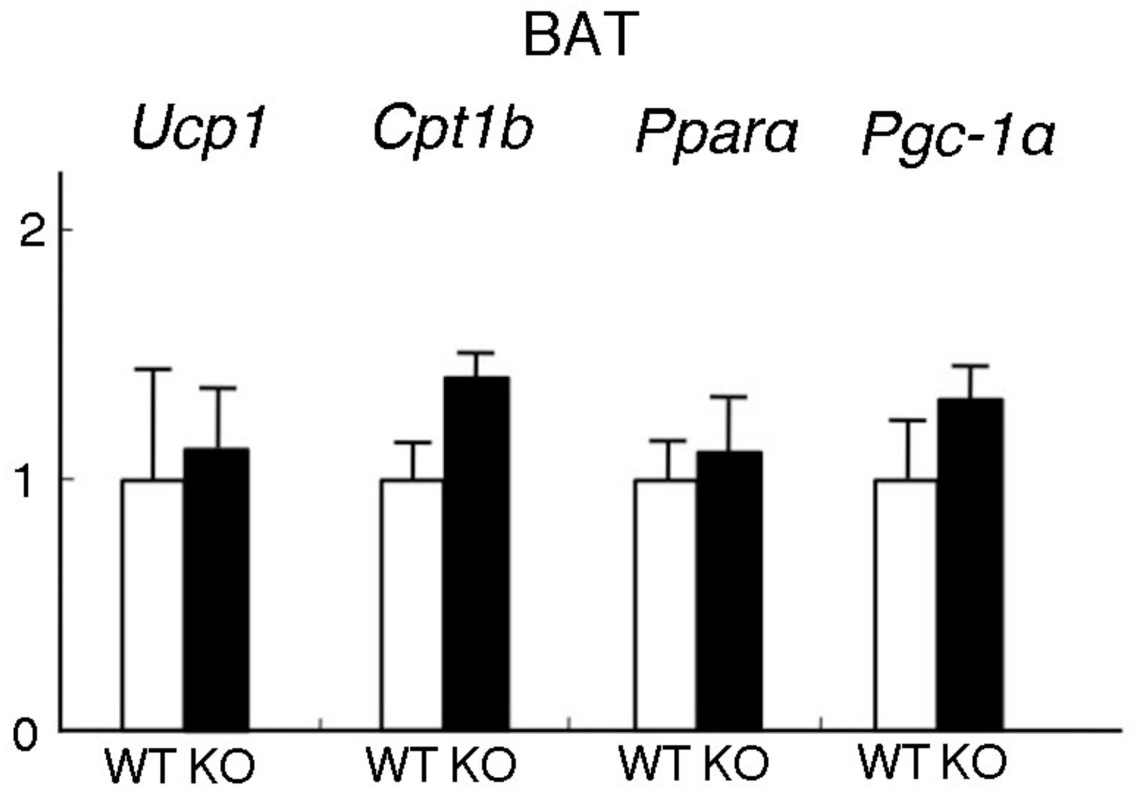

Figure S4. The expression levels of thermogenic genes in BAT of WT and *neudesin* KO mice at 4 weeks of age

The expression levels of *Ucp1*, *Pgc-1α*, *Pparα*, and *Cpt1b* in the BAT of WT and *neudesin* KO mice at 4 weeks of age were measured (n=3-5). *18S* rRNA levels were used as an internal control and expression levels in WT mice were taken as 1.0.

Figure S5

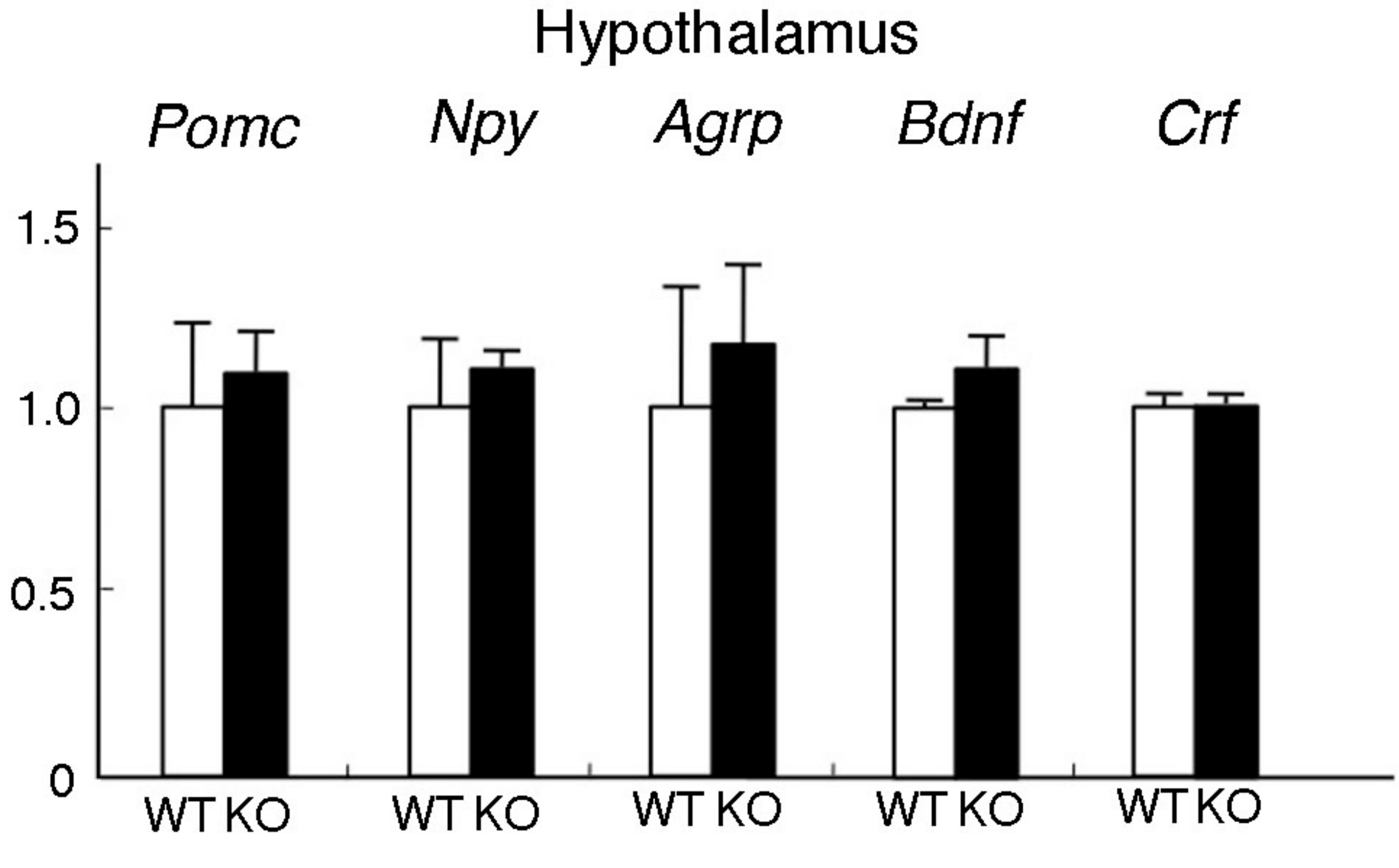

Hypothalamus
Pomc
Npy
Agrp
Bdnf
Crf
1.5
1.0
0.5
0
WT KO
WT KO
WT KO
WT KO
WT KO

Figure S5. The analysis of neuropeptides expression in the hypothalamus of WT and *neudesin* KO mice fed HFD

The expression levels of *Pomc*, *Npy*, *Agrp*, *Bdnf*, and *Crf* in the hypothalamus of WT and *neudesin* KO mice fed HFD at 16 weeks of age. *18S* rRNA levels were used as an internal control and expression levels in WT mice were taken as 1.0 (n=3-7).

Figure S6

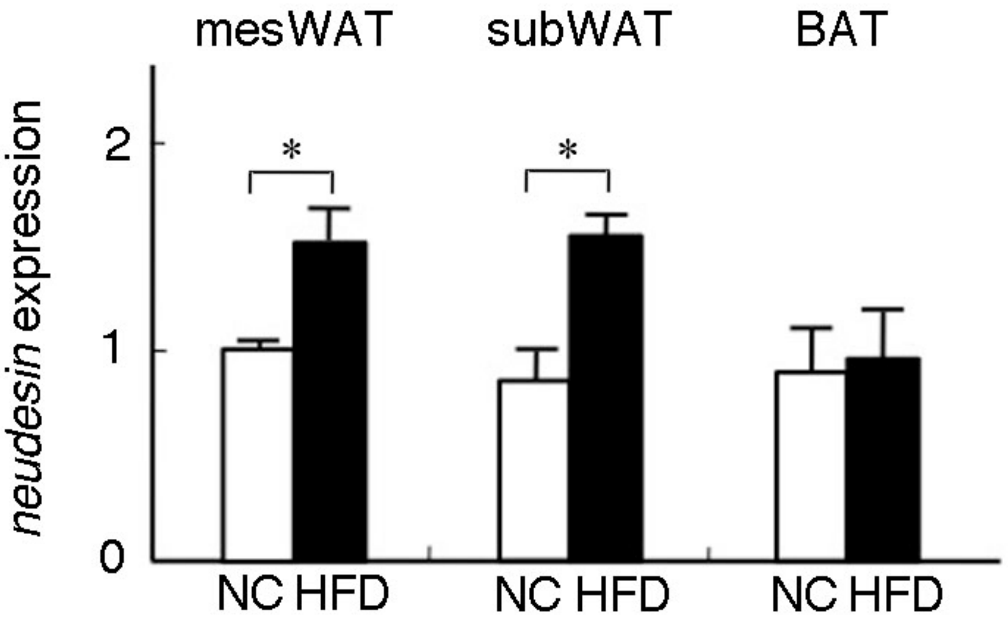
mesWAT
subWAT
BAT
neudesin expression
2
1
0
*
*
NC HFD
NC HFD
NC HFD

Figure S6. The analysis of *neudesin* expression in the WAT and BAT of WT mice fed NC or HFD

The expression levels of *neudesin* in the mesWAT, subWAT, and BAT of WT mice fed NC or HFD at 16 weeks of age. *18S* rRNA levels were used as an internal control and expression levels in the mesWAT of WT mice fed NC were taken as 1.0 (n=3-7; *, $p<0.05$).
